# FDA's insights: implementing new strategies for evaluating drug-induced QTc prolongation

**DOI:** 10.1007/s10928-025-09985-4

**Published:** 2025-06-25

**Authors:** Yanyan Ji, Lars Johannesen, Christine Garnett

**Affiliations:** Division of Cardiology and Nephrology, Office of New Drugs, Center for Drugs Evaluation and Research, U.S. Food and Drug Administration, Silver Spring, USA

**Keywords:** Thorough QT study, ICH E14 guideline, QTc interval prolongation, Electrocardiogram

## Abstract

The questions and answers (Q&A) document for ICH E14/S7B provides the following advancements for QTc assessment: concentration-QTc modeling (C-QTc) as the primary analysis, accepting alternative approaches (Q&A 5.1 and 6.1) to thorough QT (TQT) studies, and incorporating an integrated nonclinical risk assessment as supporting evidence. Based on QT study reports reviewed by the FDA between 2016 and 2024, changes to the E14 guideline have resulted in a 34% decrease in the proportion of TQT studies, while the use of C-QTc analysis as the primary analysis has significantly increased. Studies using C-QTc instead of by-time analysis as the primary analysis reduced median sample sizes by 67%, 42%, and 35% for parallel, nested crossover, and crossover studies, respectively. The white paper C-QTc model was used for 60% of drugs that prolonged the QTc interval. From 2020 to 2024, reviews incorporating an integrated nonclinical risk assessment have also increased. The advancements in QTc assessments have streamlined QTc assessment and made clinical trials less resource-intensive. As the advancements continue to evolve the drug safety evaluation is likely to become even more adaptive and enable more precise and targeted QTc assessment.

## Introduction

The International Council for Harmonization of Technical Requirements for Pharmaceuticals for Human Use (ICH) serves as a unique forum that brings together regulatory agencies and pharmaceutical industry representatives to discuss scientific and technical aspects of pharmaceuticals and develop ICH guidelines. ICH working groups, including representatives from regulatory agencies, meet regularly to discuss issues in harmonizing requirements. The ICH E14 guideline was developed to address the potential for drug-induced prolongation of the heart rate corrected QT (QTc) interval, which is linked to a heightened risk of torsades de pointes (TdP), a potentially fatal arrhythmia. The history of the ICH E14 guideline is rooted in efforts to improve the cardiovascular safety of new drugs. In May 2005, the ICH E14 guideline, titled “The Clinical Evaluation of QT/QTc Interval Prolongation and Proarrhythmic Potential for Non-Antiarrhythmic Drugs,” was implemented by the Food and Drug Administration (FDA) for most new drugs with systemic bioavailability [1]. The guideline provided a framework for conducting thorough QT (TQT) studies, which became the standard at the time for assessing a drug’s impact on the heart rate corrected QT (QTc) interval. These studies in healthy volunteers were designed to exclude a 10-millisecond (ms) threshold in the mean baseline- and placebo-corrected QTc interval (∆∆QTc) and included a positive control arm to ensure the sensitivity of the results. A 10-ms increase in mean ∆∆QTc in healthy subjects is not considered proarrhythmic but could be precarious with further augmentation by increased drug exposure in patients with underlying cardiac risk factors. Therefore, a drug found to prolong the mean ∆∆QTc interval greater than 10 ms in a TQT study would typically warrant further ECG monitoring and safety evaluation in clinical trials to evaluate its proarrhythmic risk in the targeted population. The utilization of concentration-QTc modeling further enhanced our interpretation of the data from TQT studies, allowing for the evaluation of clinically relevant scenarios, other than those tested under controlled conditions [2].

The E14 guideline applies mostly to small molecules. Products such as targeted proteins and monoclonal antibodies (mAb) are generally considered to have low likelihood of direct ion channel interactions and a TQT study is not necessary, unless the potential for proarrhythmic risk is suggested by mechanistic considerations or data from clinical or non-clinical studies [3, 4].

The ICH E14 guideline has been updated periodically to address emerging questions during its implementation across regulatory regions. In 2008, the ICH released its first Question and Answer (Q&A) document to provide additional guidance on certain aspects of TQT studies and the interpretation of study results. The Q&A document has been further revised to incorporate new insights and advancements in methodologies for assessing QTc prolongation [5]. One of the major evolutions in the guideline has been the acceptance of alternative approaches to a TQT study. Another major evolution in the guideline has been the use of an integrated nonclinical risk assessment as important support for alternative approaches to replace TQT studies [5]. The important sections in E14 and Q&As relevant to this manuscript are described in Table 1 and the differences in E14 approaches are described in Table 2.

**Table 1:** Key sections of ICH E14 and S7B question & answer documents

Guideline and Sections	Topic	Description	Examples
E14 [1]	TQT study	Provides recommendations concerning the design, conduct, analysis, and interpretation of TQT studies. Key components of a TQT study include: • Positive control: Incorporation of a known QT-prolonging drug (e.g., moxifloxacin) to demonstrate assay sensitivity. This element ensures that the study can detect small mean increases in the QTc interval • Placebo control: Incorporation of a placebo arm to account for natural variability in QTc intervals. This allows researchers to distinguish drug-induced effects from background fluctuations • Dose/Exposure: Incorporation of a dose that covers the expected increase in drug concentration due to intrinsic or extrinsic factors (high clinical exposure). This approach helps evaluate the drug's cardiac safety profile under conditions of increased exposure, which may occur in clinical practice	[24, 25]
E14 Q&A 5.1 [5]	Substitute for TQT study and concentration-response analysis	Provides recommendations on use of concentration-QTc analysis of early clinical trial data as a substitute for TQT studies. Studies conducted under this approach include: • No positive control: Unlike TQT studies, this approach does not require the inclusion of a known QT-prolonging drug. Instead, QTc interval are assessed over a range of exposures exceeding high clinical exposures • Placebo control: Incorporation of a placebo arm to account for natural variability in QTc intervals, similar to TQT studies • Dose/Exposure: Incorporation of doses to cover drug exposures up to 2 times the high clinical exposure to waive the need for a positive control • Integrated nonclinical risk assessment: Used when clinical trial doesn't reach 2 times high clinical exposure This 5.1 approach offers an alternative to traditional TQT studies, potentially allowing for more efficient cardiac safety assessments during early-phase clinical development	[10, 26, 27]
E14 Q&A 6.1 [5]	Alternative QT study	This approach is typically used when standard QTc evaluation methods are not practical or ethical, which is often the case with oncology products due to the nature of the disease and treatment. The key features of this alternative approach include: • No positive control: Not required to demonstrate assay sensitivity. However, this absence, especially when combined with the lack of high exposures for QTc assessment, creates reluctance to conclusively determine the absence of a QTc effect • No placebo control: A placebo arm is often not included, which is often more appropriate for oncology trials where withholding active treatment may be unethical • Dose/Exposure: Incorporation of doses to cover the expected clinical exposure range. High clinical exposures may not be feasible due to the safety profile of the drug • Integrated nonclinical risk assessment: A"double-negative"assessment can be used as supplementary evidence. The in vivo study should have sensitivity similar to dedicated clinical QT studies. This additional assessment helps to further characterize the drug's cardiac safety profile, compensating for limitations in the clinical trial (no positive and placebo controls, no supratherapeutic doses)	[26]
S7B Q&A 1 [5]	Integrated nonclinical risk assessment	General strategy for use nonclinical information as part of an integrated risk assessment. A “double-negative” assessment is characterized by two key negative findings: • hERG safety margin: The drug should have a hERG safety margin higher than that of drugs known to cause QTc prolongation. This is determined through in vitro IKr/hERG assays • In vivo QTc assessment: The drug should show no QTc prolongation in an in vivo assay of sufficient sensitivity, conducted at exposures that exceed clinical levels Both components of this assessment, the in vitro hERG assay and the in vivo QT assay, should be conducted according to best practice recommendations as defined in ICH S7B Q&As 2 and 3. This"double-negative"assessment provides a higher level of confidence in the drug's low risk for QTc prolongation, as it combines two different but complementary methods of assessment	[26]

**Table 2:** Key features of TQT studies and alternative QTc assessment

Type	Positive control	Placebo control	Preferred Exposure coverage	Criteria to Claim Negative Study	Double-Negative Integrated Nonclinical Risk^1^	Overall Interpretation
TQT Study	Single dose oral moxifloxacin, 400 mg	Required	≥ High clinical	• ∆∆QTc UB 95% CI < 10 ms at high clinical exposure using by-time or C-QTc analysis • Assay sensitivity established by positive control	Optional	No clinically significant QTc prolongation
5.1 Approach	Optional	Required	≥ 2xHigh clinical	∆∆QTc UB 95% CI < 10 ms at high clinical exposure using C-QTc analysis	Optional	
			≥ High clinical		Required	
6.1 Approach	Optional	Optional	≥ Clinical	∆QTc UB 95% CI < 10 ms at clinical exposure using by-time or C-QTc analysis	Optional	A mean increase in the QTc interval ≥ 20 ms is unlikely
					• Required, and includes an in vivo assay with similar sensitivity to a TQT study	Low proarrhythmic risk due to delayed repolarization^2^

Abbreviations: CI, confidence interval; C-QTc, concentration-QTc; UB, one-sided upper bound; ∆∆QTc, baseline and placebo-adjusted QTc; ∆QTc, baseline-adjusted QTc

^1^Double Negative integrated nonclinical risk assessment includes (1) hERG assays following best practices (ICH S7B Q&A 2.1) showing low risk for parent and metabolite (ICH S7B Q&A 1.1); and (2) no evidence of QTc prolongation in an in vivo assay conducted according to ICH S7B Q&A 3 at exposures covering high clinical exposures of parent and major human metabolites

^2^Assessment also includes a cardiovascular safety evaluation that does not suggest increased rate of adverse events that signal potential for proarrhythmic effects. A low likelihood of proarrhythmic effects due to delayed repolarization could still be made for a drug that has an upper bound of the one-sided 95% confidence interval around the estimated maximal effect on ΔQTc of 10 ms or more. The determination will depend on the quality and details of the clinical and nonclinical data

Alternative approaches to the TQT study include a concentration-QTc (C-QTc) assessment in early clinical trial(s) that can serve as a TQT study substitute. QTc assessments based on data from early clinical studies (e.g., single and multiple ascending dose studies) could well suffice to demonstrate a drug has no QT liability, replacing dedicated TQT studies. This approach, described in ICH E14 Q&A 5.1 and known as the 5.1 approach, relies on early clinical trial design elements, such as placebo control, high doses that cover at least twice the expected high clinical exposure to waive the need for a positive control, and high-quality electrocardiogram (ECG) recordings. If there are any hERG-active metabolites, the highest dose should cover their high clinical exposures.

Similar to the TQT study, a negative outcome is defined as one where the upper bound of the one-sided 95% confidence interval (CI) for the mean ∆∆QTc is below 10 ms at the highest clinically relevant plasma concentration using C-QTc analysis. If clinical trials do not meet the exposure multiple, a “double-negative” integrated nonclinical risk assessment can be used as supplementary evidence, including both a low-risk hERG assay following best practice (S7B Q&A 2 [5]) and no QTc prolongation evidence in an in vivo assay per ICH S7B guidelines (S7B Q&A 3 [5]). The idea of using nonclinical information to support clinical QTc evaluation is supported by several studies in literature [6, 7]. The implementation of the 5.1 approach made QTc evaluation studies less resource-intensive while maintaining safety and efficacy standards. No drugs have been removed from the US market due to TdP risks since the implementation of these guidelines by the FDA [8].

If a TQT study or 5.1 approach is not feasible—for instance, due to safety concerns that prevent drug administration to healthy volunteers [9]—an alternative QT study may be considered as described in ICH E14 Q&A 6.1 (referred to as the 6.1 approach). This approach, often applied to oncology products, incorporates as many of the TQT design features as possible but generally does not require placebo or positive control, or supratherapeutic dose levels. The alternative QT study is expected to include doses that cover at least clinically relevant exposure to both the parent drug and its major metabolites. Without positive and placebo controls, conclusions about a lack of a small effect may be uncertain; however, if the upper bound of the one-sided 95% CI for the mean baseline-corrected QTc (∆QTc) is less than 10 ms, the product is unlikely to have an actual mean effect as large as 20 ms (E14 Q&A 6.1 [5]). A “double-negative” integrated nonclinical risk assessment can be used as supplementary evidence, including both a low-risk hERG assay and no QTc prolongation evidence in an in vivo assay that must have sensitivity comparable to a TQT study (S7B Q&A 3 [5]).

The aim of this paper is to summarize FDA’s experience with the various E14 approaches (Table 2). We will explore submission trends, changes in analytical methods since the introduction of ICH E14 Q&As, the impact of the white paper C-QTc model [10], and implementation of integrated nonclinical risk assessments.

## Methods

Drug, study design, E14 approach and regulatory information were extracted from sponsor-submitted QT study reports and clinical pharmacology tables, and FDA reviews between January 2016 and August 2024. We chose 2016 because the Q&A R3, in which Q&A 5.1 was revised to use concentration response modeling of QTc as the primary analysis, was approved by the ICH Assembly in December 2015 and implemented by the FDA in 2016. All QT study reports reviewed by the FDA from 2016 to 2024 that fall under the ICH E14 scope were included. Data extracted included application number, therapeutic area, E14 approach (TQT study, 5.1, or 6.1 approach) and rationale for the approach, study design (positive/placebo control; parallel, crossover, or parallel with nested crossover design), primary analysis method (by-time or C-QTc analysis), study size, and study outcome. For positive studies, we also extracted information about the type of C-QTc model (white paper model [10] or other pharmacodynamic models).

To evaluate the experience with the integrated nonclinical risk assessment, we extracted information from reviews of alternative approaches of sponsor’s submissions between 2020, the year of the release of the draft ICH E14/S7B Q&A document, which introduced the concept of an integrated nonclinical risk assessment, and 2024. The use of nonclinical data to inform QTc prolongation risk in FDA reviews before 2020 had significantly different methodology and interpretation from the integrated approach introduced in 2020. Therefore, nonclinical data predating 2020 were not included in this evaluation. For each submission and review, we extracted information about the E14 approach and its associated limitations (including clinical, in vitro, or in vivo assessments). It is important to note that the scope of this analysis did not encompass tracking the frequency with which nonclinical data translate into clinical findings. Specifically, we did not evaluate how often drugs demonstrating affinity for hERG or exhibiting QTc prolongation in dogs or non-human primate models subsequently led to significant QTc prolongation within the proposed therapeutic exposure range in human subjects.

For all information extracted the cutoff date was July 31_,_ 2024.

Data were analyzed and visualized using R (version 4.3) and RStudio (version: 2023.12.1).

## Results

### Trends in E14 approaches from 2016 to 2024

**Fig. 1:**
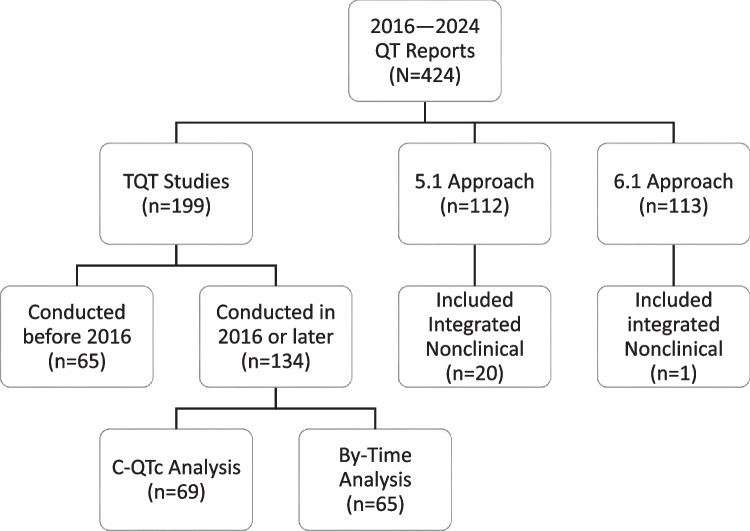
Summary of 424 QT study reports reviewed by FDA between 2016 and 2024

A summary of the QT study report database is shown in Fig. 1. FDA reviewed a total of 424 QT study reports between 2016 and August 2024 ― 47% were TQT studies, 26% were studies following the 5.1 approach, and 27% were studies following the 6.1 approach.

Under the 6.1 approach, 79% were for oncology indications. Other indications that were evaluated under the 6.1 approach included opioid use disorders (3%), malaria (2%), and rare kidney diseases (2%). The proportion of oncology indications and non-oncology indications remained stable over the years.

**Fig. 2:**
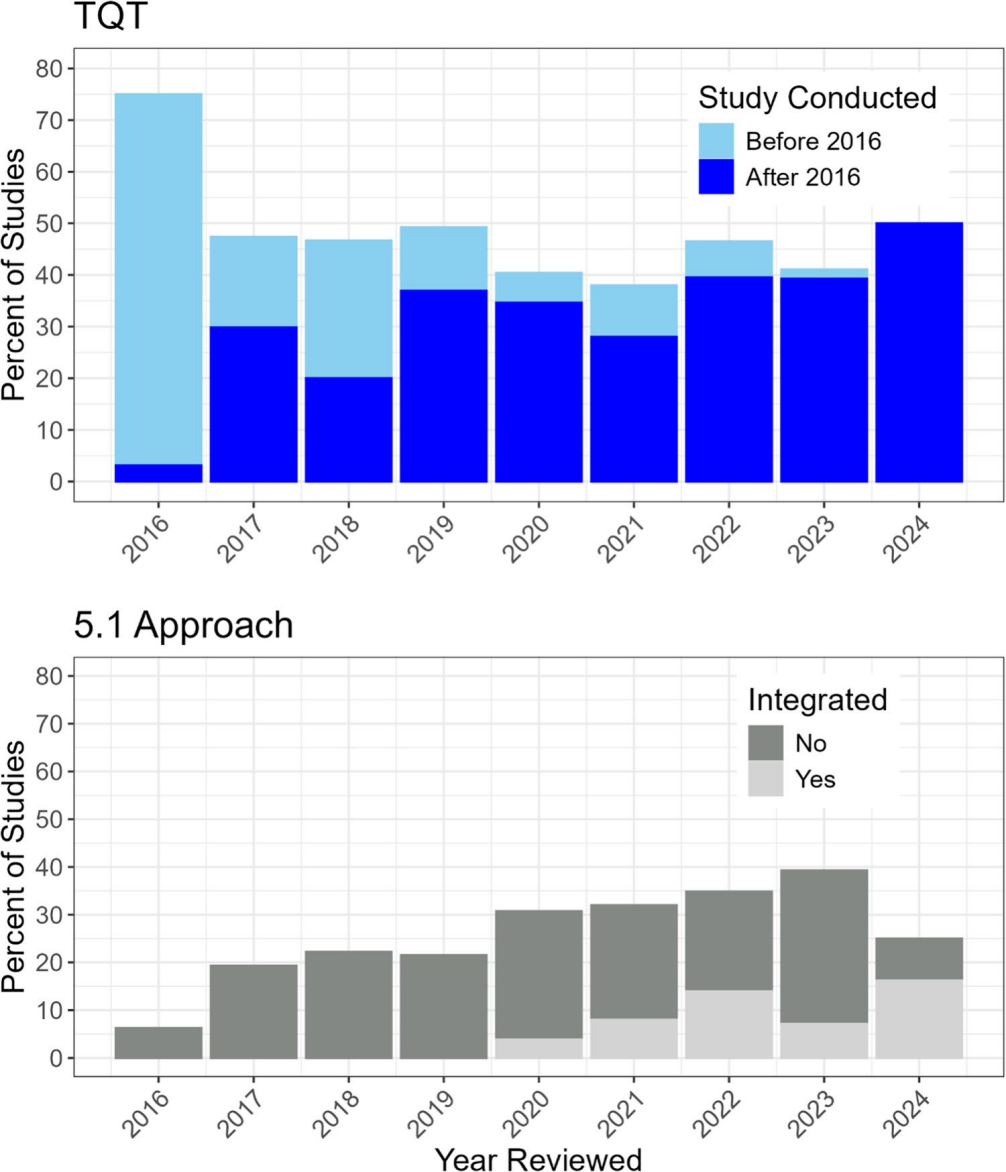
Percentage of TQT studies (Top) and studies following the 5.1 approach (bottom) by the year reviewed by FDA

When comparing TQT studies and studies following the 5.1 approach by the year that the studies were reviewed by the FDA, the percentage of TQT studies has decreased from 75% of all QT study reports in 2016 to 41% in 2023 and the percentage of assessments performed under the 5.1 approach has increased from 6% in 2016 to 39% in 2023 (Fig. 2). Overall, the introduction of E14 Q&A 5.1 caused a reduction in the percentage of TQT reports by 34%. Among the 134 TQT studies conducted in 2016 or later, the sponsor did not request to use a 5.1 approach in 72% of cases. Of the 38 cases where the sponsor did request a 5.1 approach, the FDA did not agree in 36 cases. The most common reason was insufficient exposure coverage to waive the positive control. Other reasons included study design limitations (such as lack of placebo) and inadequate ECG quality. There were two applications where the FDA agreed with a 5.1 approach, but the sponsor still conducted a TQT study. Since our analysis focused on QT study reports, we did not systematically track the frequency with which QTc assessment requests utilizing the 5.1 approach was not accepted by the FDA. This limitation in our data collection methodology should be noted when interpreting the results of our analysis.

**Fig. 3:**
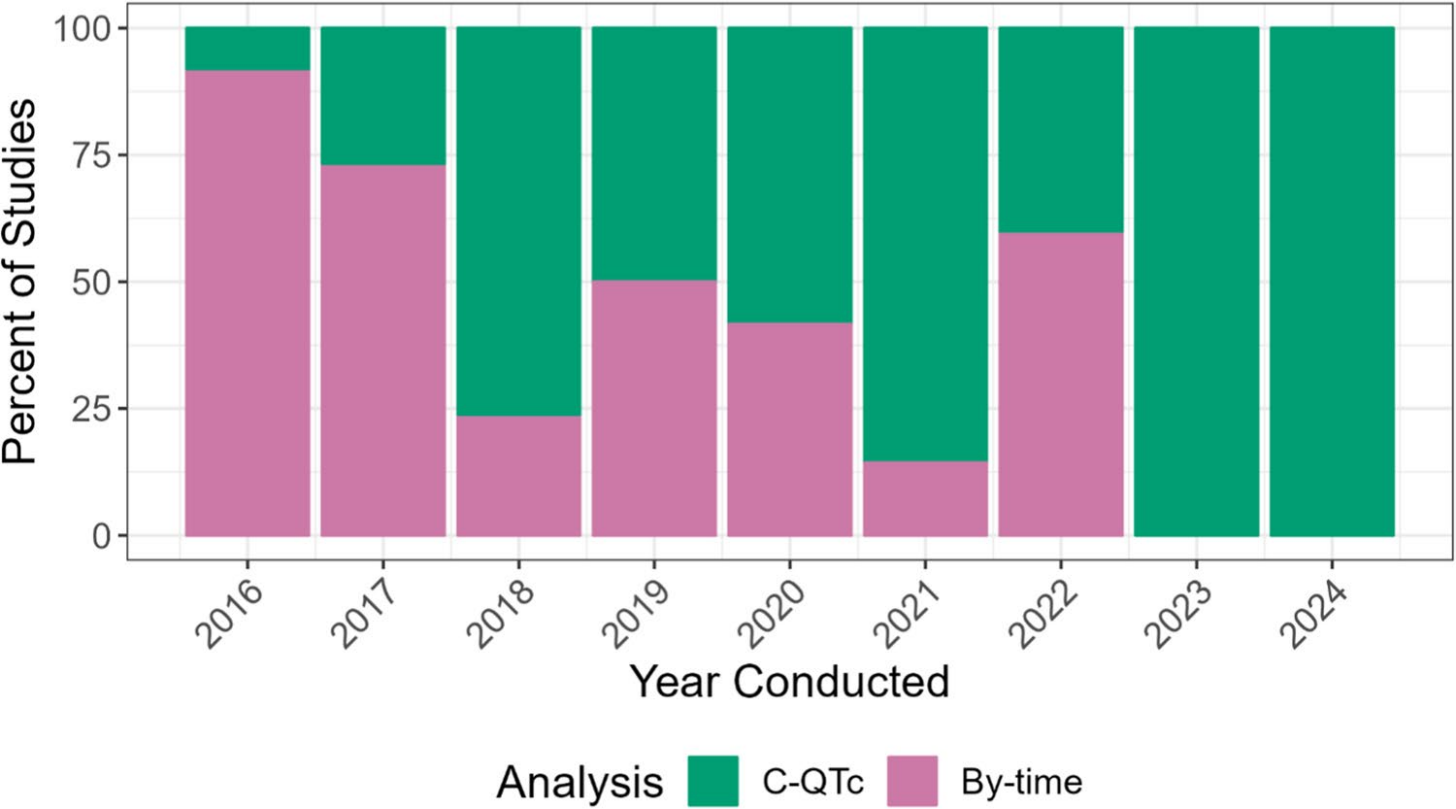
Percentage of analysis methods of TQT studies by the year study conducted

**Fig. 4:**
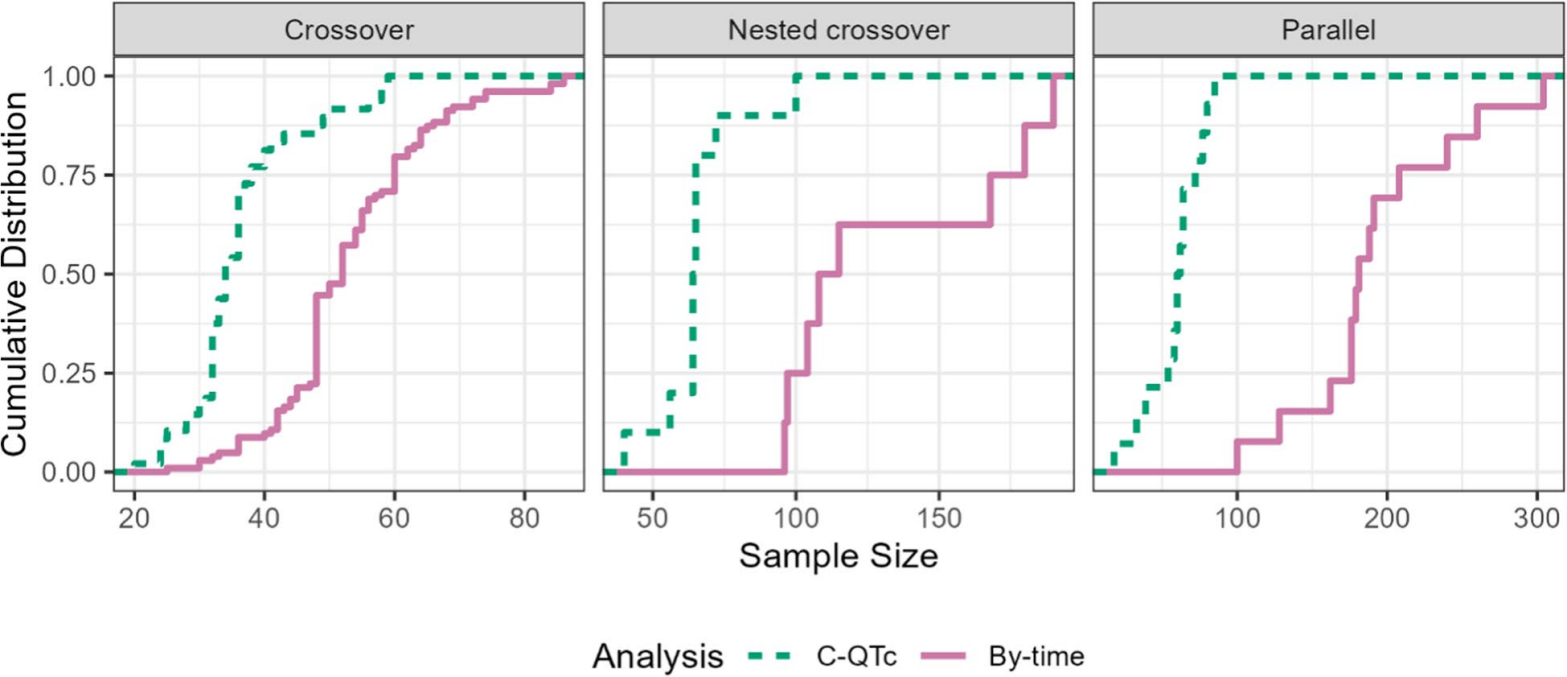
Cumulative distribution of sample size by analysis methods and study designs

While the introduction of the 5.1 approach did not fully eliminate TQT studies, it did make the studies more efficient. When examining TQT studies by the year the study was conducted, the majority of the TQT studies used C-QTc analysis as the primary analysis method by 2018 (Fig. 3). Studies that used C-QTc as the primary analysis utilized fewer participants compared to those using by-time analysis across all study designs, reducing median sample size by 67% in parallel studies, 42% in nested crossover studies, and 35% in crossover studies (Fig. 4).

### Sufficiency of the white paper C-QTc model

Of the 424 QT study reports, 70 reports involved drugs that prolonged the ∆∆QTc interval. A C-QTc modeling approach was conducted for 76% of these drugs. The main reasons for not using the C-QTc model were due to the presence of pharmacokinetic-pharmacodynamic hysteresis (14%) or the lack of a monotonic relationship between concentration and QTc (9%).

The white paper model [10] was used to describe the C-QTc relationships in 60% of the 70 QTc prolongers, whereas nonlinear models were used in 16% of the cases. The most common nonlinear models were log-linear (6%) and Emax models (4%). The C-QTc models used the observed drug concentrations of the parent moiety in 61% of the cases, both parent and metabolite concentrations in 11% of the cases, and metabolite concentrations only in 3% of the cases. When both parent and metabolite concentrations were used, multivariant C-QTc models were applied in 2 cases.

### Experience with integrated nonclinical risk assessment

An integrated nonclinical risk assessment was considered as supplementary evidence by the FDA for alternative approaches in 51 reviews between 2020 and 2024 ― 48 supported the 5.1 approach and 3 supported the 6.1 approach. There has been an increase in the annual number of reviews including an integrated nonclinical risk assessment from 2020 to 2024. The largest increase was observed from 2021 to 2022, which coincided with the implementation of the final ICH E14/S7B Q&As in 2022.

Due to the limited submissions under the 6.1 approach, we only include further statistics for submissions using the 5.1 approach with integrated nonclinical risk assessment as supplementary evidence. No limitations were identified for 46% of the reviews. Limitations were identified in 50% of reviews in the clinical QTc data and in 33% of reviews for the in vitro assay. There were no reviews that described limitations with the in vivo assessment. Limitations identified include the lack of clinical exposure in the clinical study and significant deviations in the hERG assay such as lack of reference drugs or positive controls.

## Discussion

Over the past two decades, there have been significant changes in the way drug-induced QTc prolongation has been assessed, transforming both the clinical methodology and regulatory expectations. Drug-induced QTc prolongation, a measure of a drug’s potential to delay repolarization, has traditionally been a critical focus in the evaluation of new pharmaceuticals, especially small-molecule drugs with systemic bioavailability. Not all drugs, however, particularly those with low systemic exposure, require dedicated QT studies as per ICH E14 guidelines. For some drugs, especially those with known QTc effects, intensive ECG monitoring in later clinical phases may be preferred over a dedicated QT study. QT evaluations have expanded to include TQT studies, C-QTc analyses in early trials, and tailored QTc assessment based on drug-specific profiles (e.g., large targeted proteins) and therapeutic contexts. Meanwhile, pharmaceutical companies have improved at avoiding molecules likely to block the human ether-a-go-go-related (hERG) channel, the most common cause of QTc prolongation. However, there remain concerns within the scientific communities that intensified global focus on QTc safety may have unintended consequences such as the selection of suboptimal therapeutic agents, the abandonment of promising drug pathways, or the inadvertent oversight of other significant toxicities.

Initially, the TQT study was resource-intensive, incorporating multiple treatment arms, and requiring large sample sizes. A need clearly existed to reduce the sample size and make the study more efficient. In 2006, the FDA established the QT Interdisciplinary Review Team, which consisted of experts from the offices of Clinical Pharmacology, Biostatistics, and New Drug. Its primary function was to conduct comprehensive reviews of the nonclinical and clinical data packages related to QTc prolongation for most new drugs following the E14 guideline. This team ensured consistency in TQT study reviews and quickly recognized the importance of C-QTc analysis as a key method for interpreting TQT studies [2].

The C-QTc relationship, which examines the relation between drug concentration and QTc prolongation, has become increasingly prominent in QTc assessments. By utilizing C-QTc analysis, investigators can predict QTc effects at various clinically relevant concentration levels. Regulatory authorities globally implemented this approach in 2015, following the ICH’s release of E14 Q&A (R3) document endorsing C-QTc modeling as a primary analysis. This innovation led to two major outcomes in clinical development: sponsors could assess QTc effects during early-phase clinical trials, serving as a substitute for traditional TQT studies, and the TQT studies themselves became more efficient. The white paper on C-QTc modeling provided methodological recommendations, including study design, prespecified linear C-QTc model and reporting standards to support regulatory submissions [10]. Since its implementation, C-QTc modeling has reduced the number of participants required in TQT studies by up to 67%, depending on study design, while maintaining robust data on cardiac safety.

The 5.1 approach, which allows C-QTc modeling in early studies as a substitute for TQT studies, has been rapidly adopted by the pharmaceutical industry. The adoption of this approach has resulted in a notable reduction in TQT study submissions to the FDA, dropping from 75% in 2016 to 47% in 2017, with levels remaining steady in the following years. The observed decrease in TQT studies can be attributed to the increased use of both the 5.1 and 6.1 approaches. Notably, while the total number of submissions increased during this period, the volume of TQT-specific studies remained relatively stable. This shift reflects a broader trend towards more flexible and efficient assessment methods in drug development. One ongoing challenge, however, is achieving sufficient exposure levels in early-phase trials, a factor that has limited further reductions in the number of TQT studies.

In 2022, new Q&As for E14/S7B were implemented, allowing a ‘double-negative’ integrated nonclinical risk assessment as supplementary evidence, enabling 5.1 submissions to cover high clinical exposure rather than attaining the high-multiple of clinically relevant exposure requirement. This adjustment has the potential to further decrease the need for TQT studies. A ‘double-negative’ integrated nonclinical risk assessment includes: (1) hERG assays following best practices (S7B Q&A 2.1 [5]) showing low risk for parent and major human metabolites (S7B Q&A 1.1 [5]), demonstrated by hERG safety margins higher than a threshold defined based on the safety margins computed under the same experimental protocol for a series of drugs known to cause TdP; and (2) no evidence of QTc prolongation in an in vivo assay conducted according to ICH S7B at exposures covering high clinical exposures of parent and major human metabolites (S7B Q&A 3 [5]). When an integrated nonclinical risk assessment is used to support a drug with low proarrhythmic risk when evaluated in an alternative QT study, the in vivo assay must have sensitivity comparable to a TQT study (S7B Q&A 3 [5]). This can be accomplished by demonstrating that the minimal detectable difference (MDD) in the assay [11] is consistent with the reported MDD from a similarly designed assay (e.g., sample size, design, animal species, ECG methodology) that showed QTc prolongation with a positive control.

The QTc assessment for nirmatrelvir as described in Table 3 illustrates how a ‘double-negative’ integrated nonclinical risk assessment was used as supplementary evidence [12]. Nirmatrelvir was approved in 2023 in combination with ritonavir (PAXLOVID) for the treatment of mild-to-moderate coronavirus disease 2019 (COVID-19) in adults who are at high risk for progression to severe COVID-19, including hospitalization or death. The recommended dose is nirmatrelvir/ritonavir 300/100 mg BID with or without food for 5 days [13]. The QTc assessment focused on nirmatrelvir because the QTc effects of ritonavir 100 mg BID have already been characterized [14]. High fat meals increase nirmatrelvir Cmax by 1.6-fold [13]. Increased exposure was observed in patients with severe renal impairment compared to healthy subjects (Cmax: ~ 1.5-fold) [13]. In the review of the QTc assessment, the high clinical exposure scenario was considered as 150 mg/100 mg (nirmatrelvir/ritonavir) in patients with severe renal impairment under fed conditions. The exposure coverage for nirmatrelvir in the QTc assessment was only 1.5-times the high clinical exposure scenario [12]. This is less than the exposure coverage necessary to waive the requirement for a separate positive control. However, the applicant included a ‘double-negative’ integrated nonclinical risk assessment as supplementary evidence. The clinical and nonclinical data therefore supported concluding an absence of QTc prolongation like a TQT study. Examples of approved drugs that used an integrated nonclinical risk assessment under the 5.1 approach are QALSODY (tofersen) [15] and WAINUA (eplontersen) [16]. So far, no drug has successfully had an integrated nonclinical assessment to support low proarrhythmic risk under the 6.1 approach.

**Table 3:** Example of double negative integrated nonclinical data that provided supplementary evidence to support the lack of QTc prolongation in a small phase 1 study of nirmaterelvir/ritonavir [12]

Case 1: Paxlovid (nirmatrelvir and ritonavir)	
E14 Approach	5.1 Approach + Integrated nonclinical risk assessment
Clinical data	The QTc effects of nirmaterelvir were assessed in a randomized, double-blind placebo-controlled crossover study in 10 healthy subjects. A supratherapeutic dose of nirmatrelvir was administered as a split dose over 4 h with 100 mg ritonavir. Study included digital, 12-lead ECG recordings and time-matched PK measurements at 0, 2, 3, 3.5, 4, 4.5, 5, 5.5, 6, 8, 12, 24, 48, 72 and 96 h post dosing. The mean C_max_ covers 1.5 times the anticipated high clinical exposure in subjects with severe renal impairment under fed condition
Primary analysis	The white paper C-QTc model was used as primary analysis. The upper bounds of the two-sided 90% CI for ∆∆QTcF for therapeutic and high clinical exposure scenario were below 10 ms regulatory threshold
hERG assay	• hERG assays met most of the best practice recommendations • hERG results showed that nirmatrelvir has a hERG safety margin of > 44x (12% inhibition at 300 µM, the highest tested concentration). The estimated IC50 of nirmatrelvir on hERG current are from 1158 µM to 18266 µM with the safety margin from 173 × to 2726 × by fitting data to hill equation with a hill slope from 1.5 to 0.5, respectively • Three reference drugs dofetilide, ondansetron, and moxifloxacin have hERG safety margins of 59x, 3 × and 22x, respectively. The estimated safety margin of nirmatrelvir is larger than the safety margins of dofetilide, ondansetron, and moxifloxacin • Overall, nirmatrelvir has a low risk for QTc prolongation by directly inhibiting the hERG current at high clinical exposure
In vivo assay	No QTc prolongation was observed at exposures twice the anticipated high clinical exposure in the in vivo primate study which was conducted according to best practice recommendations
Overall conclusion	This clinical and integrated nonclinical risk assessment can be used as a substitute for a TQT study under ICH E14 Q&A 5.1

The white paper C-QTc model is a prespecified linear mixed-effect model that is used primarily to exclude a 10-ms mean increase in ∆∆QTc as measured by the upper bound of the one-side 95% confidence interval [10]. This simple model is recommended because it can be used for most study designs supporting QTc assessments, such as single ascending dose (SAD), multiple-ascending dose (MAD), and TQT studies. The linear model has been shown to have robust operating characteristics in simulation studies [17] and in a prospective, proof-of-concept study of five QTc prolonging drugs [18]. In our experience, for 70 drugs that were found to prolong the QTc interval, the linear white paper model was used for 60% of the reports to characterize the QTc effects.

Mechanistic insights into QTc prolongation have also shaped these evolving methods. For instance, drugs that inhibit the hERG channel generally show a linear C-QTc relationship, whereas a nonlinear C-QTc relationship often indicates a different underlying mechanism or inhibition of multiple cardiac ion channels. Caution in using nonlinear models without a solid mechanistic rationale was highlighted in the case of macimorelin [19].

The macimorelin case illustrates the challenges with predicting the QTc changes at doses not studied when the mechanism for QTc prolongation is unknown [19]. The TQT study was a 3-way cross-over study with a single supratherapeutic dose. An increase in the QTc interval was observed following a single dose; however, the increase in QTc was delayed relative to macimorelin concentrations. The mechanism for the observed delay was unknown. An effect-compartment could be used as a model for such data, which would suggest less QTc prolongation at lower doses. While reviewing the TQT study, the FDA reviewer evaluated a SAD study of macimorelin as well, in which serial ECG/PK data were collected at 3 dose levels, with the highest dose being the same as the single dose from the TQT study. A similar delay and magnitude of QTc prolongation were observed for the overlapping dose (Table 4). However, the time-course for the QTc interval for the two lower doses were identical to that of the high dose. This observation therefore challenges the use of a simple effect-compartment model to describe observed data. Relying on a C-QTc model based solely on the TQT study using a single supratherapeutic dose of macimorelin might have underestimated the QTc effect, underscoring the importance of dose response data in the QTc assessment if the mechanism is not mediated by hERG inhibition.

**Table 4:**
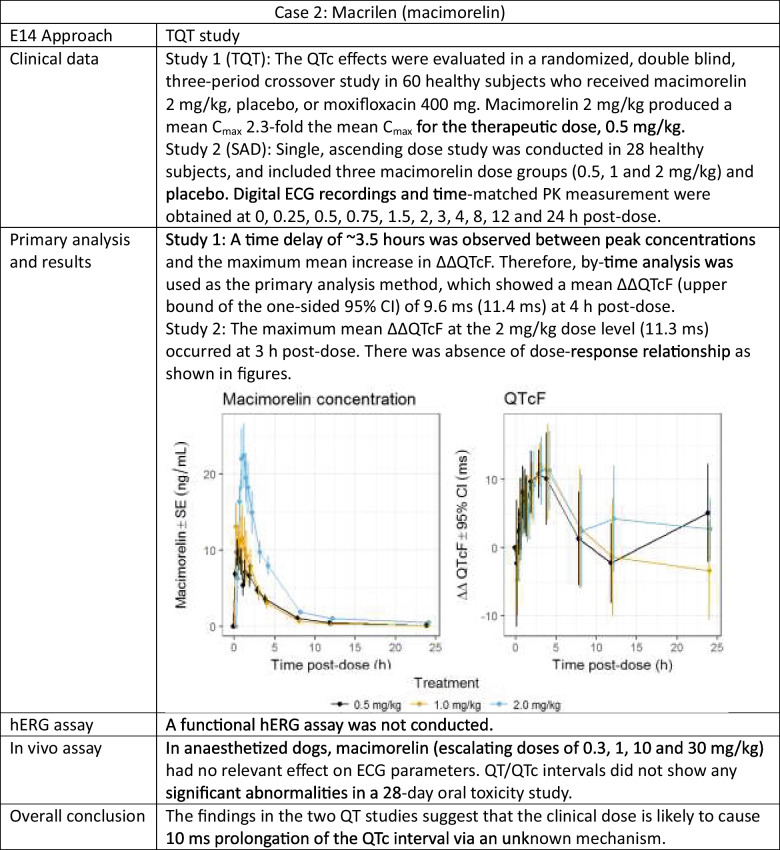
Example of QTc prolongation with unknown mechanism [19]

Challenges with using simple C-QTc models have also been observed for other QTc prolongers such as oliceridine [20] and gepirione [21]. In both cases, apparent time-dependent QTc prolongation effect was observed. In the case of oliceridine, QTc prolongation decreased after repeat dosing due to an unknown mechanism. Two TQT studies were conducted. In the single-dose TQT study where therapeutic (3 mg IV infusion) and supratherapeutic (6 mg IV infusion) doses were tested, dose-dependent QTc prolongation (3 mg: 7 ms [upper two-sided 90% CI: 9 ms]; 6 mg: 12 ms [14 ms]), which occurred after peak oliceridine plasma concentration, was observed [22]. In the multiple-dose TQT study where intermittent dosing over 24 h to the maximum daily cumulative dose of 27 mg (maximum daily dose recommended by the label) was evaluated, QTc prolongation, which peaked at 9 h post the 1 st dose (mean QTcF 11 ms [upper two-sided 90% CI: 13 ms]), diminished after 12 h [22]. In the case of gepirone, C-QTc analysis revealed different relationships on Day 1 (non-linear) and Day 7 (linear) with less QTc prolongation on Day 7 despite higher plasma concentration. The mechanism is unknown. These challenges create opportunities for further advancement of C-QTc modeling, as supported by mechanistic data.

Various models, including in silico, in vitro, ex vivo and in vivo models, can be used as part of a comprehensive risk assessment strategy to evaluate the proarrhythmic risk of QTc-prolonging pharmaceuticals in humans. The utilization of in vitro and in silico models aligns with the 3R (reduce/refine/replace) principles, effectively reducing animal use in research. For these proarrhythmia risk prediction models to be considered valid for regulatory purposes, they must follow general principles. These include having a clearly defined endpoint consistent with the context of use, a specified domain of applicability/scope with acknowledged limitations, a mechanistic interpretation of the model, and the ability to propagate uncertainty from model input to model prediction (as outlined in S7B Q&A 4.1 [5]). These principles align with general principles of ICH M15 for model-informed drug development [23]. The implementation of these models could potentially address practical limitations in evaluating supratherapeutic doses in healthy subjects or patients and provide valuable insight into the clinical implications of small changes in QTc interval, particularly in the context of drug-drug interactions.

## Conclusions

The approach to assessing drug-induced QTc prolongation has advanced significantly, driven by a better understanding of drug mechanisms, refined regulatory guidelines, and more use of modeling techniques. The adoption of C-QTc modeling, as exemplified by the 5.1 approach, has streamlined QTc assessments, making clinical trials less resource-intensive while maintaining safety and efficacy standards. As these innovations continue to evolve, the landscape of drug safety evaluations is likely to become even more adaptive, enabling more precise and targeted risk assessments tailored to the pharmacological characteristics of each drug. This ongoing transformation not only benefits clinical research but also supports patient safety by providing a clearer and more accurate understanding of cardiac risk profiles in new and existing pharmaceuticals.

## Data Availability

Our data contain confidential information and cannot be shared openly.
